# Supplementary figures and images for: Numb provides a fail-safe mechanism for intestinal stem cell self-renewal in adult Drosophila midgut
Source: bioRxiv. 2025 Feb 15:2024.11.06.622285. Originally published 2024 Nov 7. Preprint. [Version 2] doi: 10.1101/2024.11.06.622285 (PMC11580950; doi:10.1101/2024.11.06.622285)

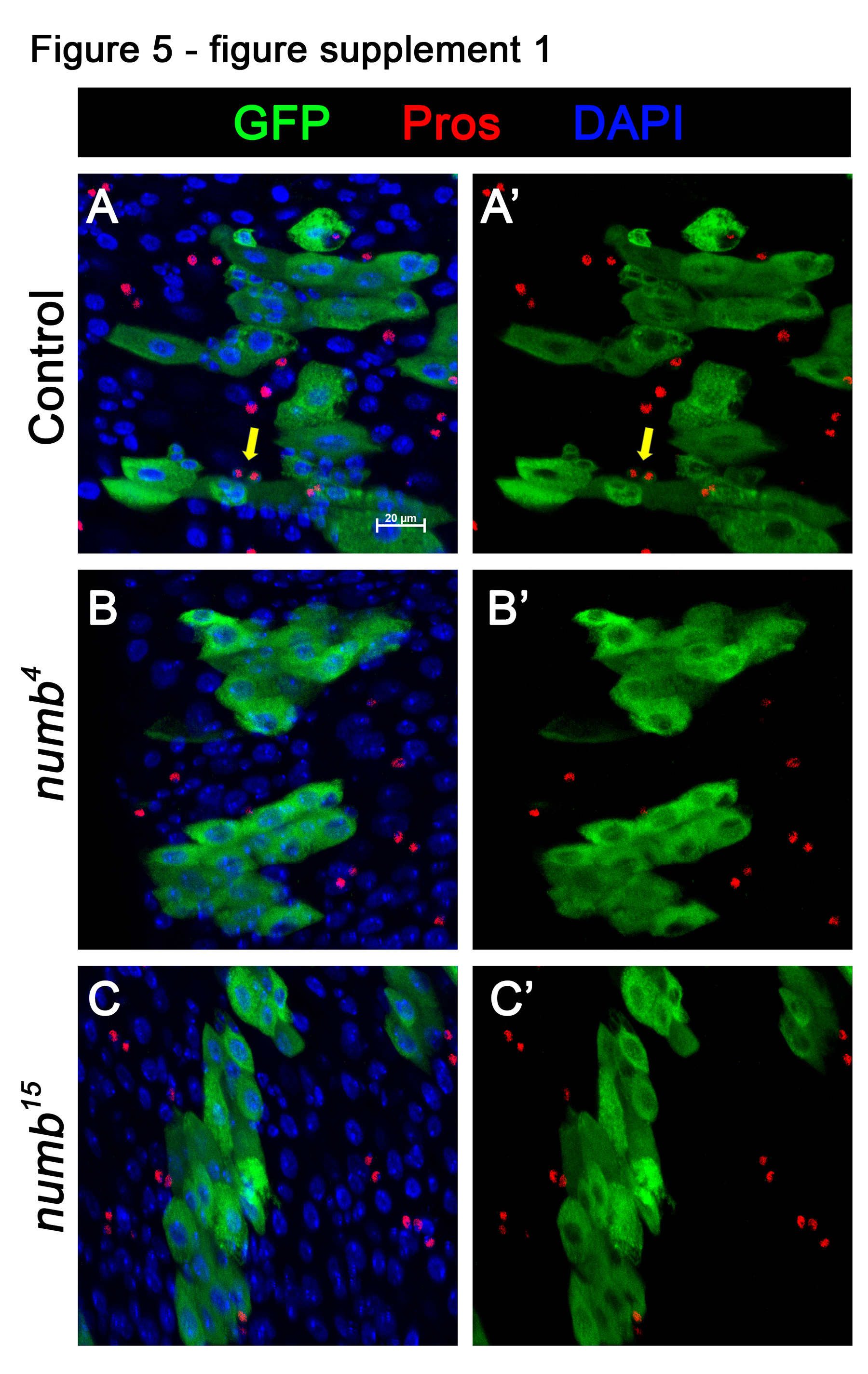

Supplement: Supplement 1 [file media-1.jpg]
