# Supplementary material for: Numb provides a fail-safe mechanism for intestinal stem cell self-renewal in adult Drosophila midgut
Source: bioRxiv. 2024 Nov 7:2024.11.06.622285. Preprint. [Version 1] doi: 10.1101/2024.11.06.622285 (PMC11580950; doi:10.1101/2024.11.06.622285)
Supplement: Supplementary file 1 [file NIHPP2024.11.06.622285v1-supplement-1.pdf]

bioRxiv preprint doi: https://doi.org/10.1101/2024.11.06.622285; this version posted November 7, 2024. The copyright holder for this preprint (which was not certified by peer review) is the author/funder, who has granted bioRxiv a license to display the preprint in perpetuity. It is made available under aCC-BY 4.0 International license.

Figure 5-figure supplement1. Numb is required for EE fate determination

(A-C') ISC MARCM clone (green) of control (A, A'), *numb*^{4} (B, B'), and *numb*^{15} (C, C') are stained for Pros (red) at 14 days after clone induction. Representative clone in control guts (A, A') contains EE cells (Pros positive), as indicated with yellow arrows. Representative clones in *numb*^{4} (B, B') and *numb*^{15} (C, C') guts do not contain any EE cells. Scale bar (20 μm) is presented in (A).
